# Short- and long-term outcomes of patients with acute myocardial infarction complicated by cardiac arrest: a nationwide cohort study 2013–22

**DOI:** 10.1093/ehjacc/zuae121

**Published:** 2024-10-23

**Authors:** Jarle Jortveit, Geir Øystein Andersen, Sigrun Halvorsen

**Affiliations:** Department of Cardiology, Sørlandet Hospital Arendal, Box 416, Lundsiden, 4604 Kristiansand, Norway; Department of Cardiology, Oslo University Hospital Ullevaal, Box 4956 Nydalen, 0424 Oslo, Norway; Department of Cardiology, Oslo University Hospital Ullevaal, Box 4956 Nydalen, 0424 Oslo, Norway; Institute of Clinical Medicine, University of Oslo, Box 1072 Blindern, 0316 Oslo, Norway

**Keywords:** Myocardial infarction, Out-of-hospital cardiac arrest, In-hospital cardiac arrest, Long-term mortality

## Abstract

**Aims:**

To assess short- and long-term outcomes of acute myocardial infarction (AMI) complicated by out-of-hospital cardiac arrest (OHCA) or in-hospital cardiac arrest (IHCA) in a nationwide cohort.

**Methods and results:**

Cohort study of AMI patients admitted to hospitals in Norway 2013–22 registered in the Norwegian Myocardial Infarction Registry. Outcomes were in-hospital and long-term mortality. Cumulative mortality was assessed with the Kaplan–Meier and the life-table methods. Cox regression was used for risk comparisons. Among 105 439 AMI patients (35% women), we identified 3638 (3.5%) patients with OHCA and 2559 (2.4%) with IHCA. The mean age was 65.7 (13.2), 70.9 (12.6), and 70.7 (13.6) years for OHCA, IHCA, and AMI without cardiac arrest (CA), respectively. The median follow-up time was 3.3 (25th, 75th percentile: 1.1, 6.3) years. In-hospital mortality was 28, 49, and 5%, in OHCA, IHCA, and AMI without CA, and the estimated 5-year cumulative mortality was 48% [95% confidence interval (CI) 46–50%], 69% (95% CI 67–71%), and 35% (95% CI 34–35%), respectively. Among patients surviving to hospital discharge, no significant difference in mortality during follow-up was found between OHCA and AMI without CA [adjusted hazard ratio (HR) 1.04, 95% CI 0.96–1.13], while the long-term mortality of AMI patients with IHCA was higher (age-adjusted HR 1.31, 95% CI 1.19–1.45).

**Conclusion:**

In this large, contemporary cohort of AMI patients, in-hospital mortality of patients with OHCA or IHCA was still high. Among patients surviving to hospital discharge, long-term mortality was comparable between OHCA and AMI without CA, while the outcome of patients with IHCA was significantly worse.

## Graphical Abstract

**Graphical Abstract:**
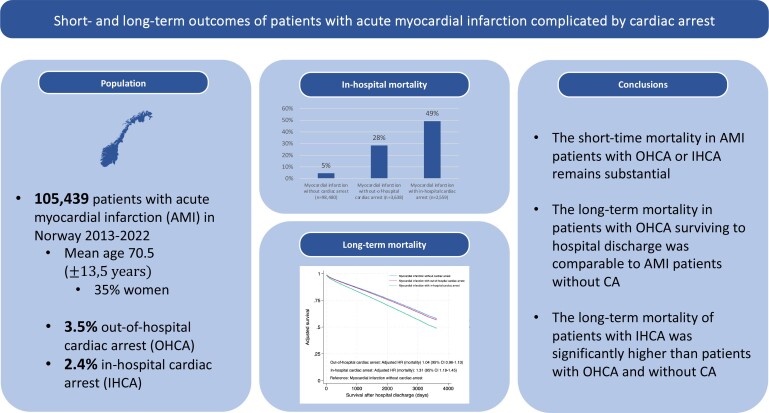

## Introduction

Out-of-hospital cardiac arrest (OHCA) and in-hospital cardiac arrest (IHCA) are infrequent but life-threatening complications to an acute myocardial infarction (AMI).^1^ Cardiac arrest (CA) together with cardiogenic shock are responsible for the majority (60–80%) of short-term mortality in AMI patients.^1,2^

Both the incidence and the mortality of AMI have declined during the last decades in Norway and other Western countries, likely attributed to improvements in coronary risk factors, increased use of acute reperfusion therapies, and increased prescription of secondary preventive therapy.^3–5^ However, little is known regarding the current incidence of OHCA and IHCA in patients with AMI, and the impact of CA as a complication to AMI on short- and long-term prognosis remains uncertain. Furthermore, starting resuscitation in the very old may be subjected to ethical considerations, and increased knowledge about the outcome of resuscitated very old AMI patients with CA is warranted.^6^

The present nationwide cohort study aimed to assess the short- and long-term outcomes of patients with AMI complicated by OHCA or IHCA in Norway in the period from 2013 to 2022.

## Methods

### Data sources

The Norwegian Myocardial Infarction Registry (NORMI) serves as a national quality register of all patients with AMI admitted to hospitals in Norway, and registration into this registry is mandatory for all hospitals (the Norwegian Cardiovascular Disease Registry Regulation and the Norwegian Health Register Act). The Norwegian Myocardial Infarction Registry encompasses demographic information such as gender and age and information on cardiovascular risk factors, previous diseases, and medication, presenting symptoms, clinical findings, electrocardiogram (ECG), in-hospital interventions and complications, and outcomes including mortality. The registration and quality of the information in the registry have been described previously.^7,8^

Information on causes of deaths was obtained by linkage of NORMI to the Norwegian Cause of Death Registry, which contains information on time and causes of all deaths in Norway (https://helsedata.no/en/forvaltere/norwegian-institute-of-public-health/norwegian-cause-of-death-registry/).

### Study population

This cohort study included all patients in Norway hospitalized with AMI from 1 January 2013 to 31 December 2022 and registered in the NORMI. For patients experiencing more than one AMI during the study period, only data from the first AMI (index myocardial infarction) were used for this analysis. For the purpose of this study, the patients were divided into the following three categories: AMI patients without CA, AMI patients with OHCA, and AMI patients with IHCA.

### Definitions

The diagnosis of AMI was made by the treating physician and was based on a combination of clinical information, repetitive ECGs, biochemical analyses, and information from coronary angiography and non-invasive imaging. With respect to the sub-classification into ST-elevation myocardial infarction (STEMI), this was, in general, based on ECG at presentation. However, due to the low specificity of ECG changes shortly after CA and return of spontaneous circulation (ROSC), a patient with CA was not classified as STEMI based on the ECG alone, but the diagnosis was made based on the combination of the information mentioned above.^9^ The NORMI adhered to the diagnostic criteria in the Third (2013–18) and Fourth (2019–22) Universal Definition of Myocardial Infarction.^10,11^ Patients with CA without evidence of acute myocardial ischaemia was not defined as AMI, and these patients were not included in this study.

The definition of OHCA was cardiopulmonary resuscitation (CPR) performed prior to hospital admission, whereas IHCA was defined as CPR during index hospitalization. Ongoing CPR at hospital admission was defined as OHCA. Patients with OHCA were categorized as OHCA regardless of any subsequent occurrence of IHCA.

### Outcomes and follow-up

The outcomes were all-cause in-hospital mortality and all-cause mortality during follow-up. Follow-up data were available through 31 December 2022.

### Statistics

Continuous variables are presented as the mean ± standard deviation (SD) or median (25th percentile, 75th percentile), and differences between groups were analyzed using independent samples *t*-tests or Mann–Whitney non-parametric tests, as appropriate. Categorical variables are presented as numbers and percentages, and differences between groups were analyzed by the χ^2^ test. Kaplan–Meier curves for survival were estimated, and differences between the groups were assessed with the log-rank test. The life-table method was used to estimate the cumulative mortality at 30 days, 1 year, and 5 years. Cox regression analyses were used to calculate hazard ratios (HRs) with 95% confidence intervals (CIs) for long-term mortality. The following covariates were included in the multivariable analyses: age, sex, smoking, previous AMI, previous stroke, previous heart failure, diabetes, antihypertensive treatment, and renal failure (estimated glomerular filtration rate [eGFR] < 60 mL/min/1.73 m^2^). Subgroup analyses investigating patients <80 years vs. ≥80 years were performed. Adjusted HRs for long-term mortality along with *P*-values for interaction between patient category and age group were calculated. The proportional hazard assumptions were checked with the proportional-hazards assumption test based on Schoenfeld residuals and log–log plot of survival. A *P*-value of <0.05 was regarded as statistically significant. The data were analysed using STATA, version 18 (StataCorp LLC, College Station, TX, USA).

### Ethics

We used existing data from Norwegian national health registries. Registration into these registries is mandatory, and consent by the patient is not required. The Regional Committee for Medical and Health Research Ethics North approved the study (REK 2016/170).

## Results

### Study population

**Figure 1:**
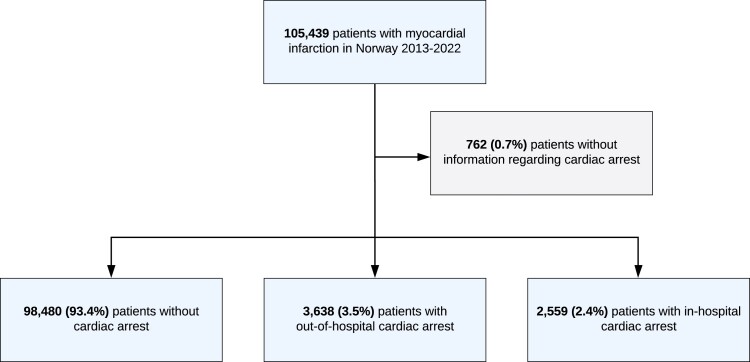
Cohort creation flow chart.

From 1 January 2013 to 31 December 2022, 105 439 AMI patients were registered into the NORMI (*Figure 1*). Information regarding OHCA, IHCA, or no CA was available in 104 677 patients. A total of 35% were women, and 29% were ≥80 years of age. We identified 98 480 (94.1%) AMI patients without CA, 3638 (3.5%) AMI patients with OHCA, and 2559 (2.4%) AMI patients with IHCA. The clinical characteristics of the three groups of patients are shown in *Table 1*.

**Table 1:** Clinical characteristics of patients with myocardial infarction with no cardiac arrest, out-of-hospital cardiac arrest, and in-hospital cardiac arrest in Norway 2013–22^a^

	Myocardial infarction without cardiac arrest	Myocardial infarction with out-of-hospital cardiac arrest		Myocardial infarction with in-hospital cardiac arrest	
	*N* = 98 480	*N* = 3638	*P* *	*N* = 2559	*P* *
Clinical characteristics					
Women (*n*)	34 733 (35.3%)	795 (21.9%)	<0.001	807 (31.5%)	<0.001
Mean age [year (SD)]	70.7 ± 13.6	65.7 ± 13.2	<0.001	70.9 ± 12.6	0.36
Age, range (year)	14–105	19–101		24–99	
Median age [year (IQR)]	71 (61,81)	66 (56,75)	<0.001	72 (63,81)	0.20
Age ≥80 years (*n*)	28 998 (29.5%)	620 (17.0%)	<0.001	711 (27.8%)	0.08
Mean body mass index (SD)	27.1 ± 4.8	27.3 ± 4.8	0.017	27.0 ± 5.0	0.30
Mean LDL cholesterol [mmol/L (SD)]	3.1 ± 1.2	3.0 ± 1.1	0.010	3.0 ± 1.2	<0.001
Smoking (*n*)	26 339 (26.7%)	1117 (30.7%)	<0.001	739 (28.9%)	0.016
First symptom cardiac arrest (*n*)	NA	1961 (53.9%)	<0.001	157 (6.1%)	<0.001
First symptom chest pain (*n*)	75 384 (76.5%)	1368 (37.6%)	<0.001	1722 (67.3%)	<0.001
Median delay from debut of symptom to hospitalization (minutes, IQR)	255 (122, 790)	102 (62, 189)	<0.001	207 (100, 718)	<0.001
Previous myocardial infarction (*n*)	19 556 (19.9%)	587 (16.1%)	<0.001	542 (21.2%)	0.10
Previous percutaneous coronary intervention (*n*)	16 061 (16.3%)	444 (12.2%)	<0.001	418 (16.3%)	0.97
Previous coronary artery bypass grafting (*n*)	8339 (8.5%)	215 (5.9%)	<0.001	239 (9.3%)	0.12
Previous heart failure diagnosis (*n*)	7110 (7.2%)	208 (5.7%)	<0.001	264 (10.3%)	<0.001
Previous stroke (*n*)	8067 (8.2%)	220 (6.0%)	<0.001	246 (9.6%)	0.010
Diabetes (*n*)	19 431 (19.7%)	585 (16.1%)	<0.001	579 (22.6%)	<0.001
Hypertension (*n*)	47 197 (47.9%)	1416 (38.9%)	<0.001	1261 (49.3%)	0.18
Renal failure (eGFR < 60 mL/min/1.73 m^2^, *n*)	28 709 (29.2%)	1120 (30.8%)	0.033	1016 (39.7%)	<0.001

Data are from the Norwegian Myocardial Infarction Registry.

SD, standard deviation; IQR, inter-quartile range; NA, not available.

^a^In patients with more than one myocardial infarction during the study period, only data from the first myocardial infarction are reported.

^*^Reference: patients with myocardial infarction without cardiac arrest.

Patients with OHCA were younger and less likely to have previous heart disease, diabetes, and hypertension compared with patients without CA. Near four of five patients with OHCA were men. Circulatory collapse was the first symptom in approximately half of the patients with OHCA, and the delay from the onset of symptoms to hospitalization was significantly shorter compared with AMI patients without CA. Out-of-hospital cardiac arrest patients were also more likely to be diagnosed with STEMI compared with patients without CA (59 vs. 26%). Patients with IHCA were of similar mean age as AMI patients without CA but were more likely to have previous comorbidities including previous heart failure, diabetes, and renal failure.

**Figure 2:**
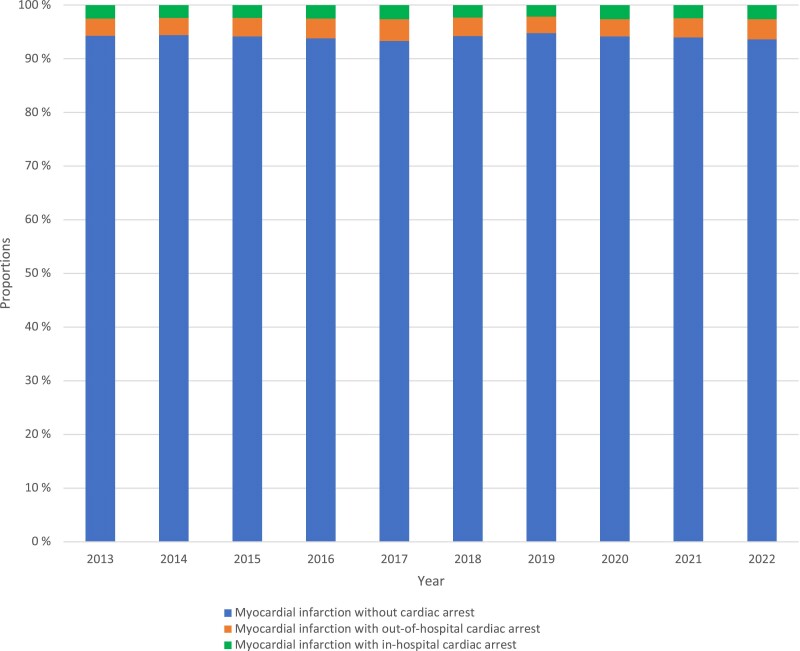
Annual proportions of patients with acute myocardial infarction without cardiac arrest, out-of-hospital cardiac arrest, and in-hospital cardiac arrest, Norway 2013–22.

The annual proportion of patients with OHCA or IHCA did not change significantly during the study period (*Figure 2*).

### Assessment and treatment

Assessment and treatment in the emergency medical service (EMS) and the hospitals are presented in *Table 2*. Compared with AMI patients without CA, patients with OHCA were more likely to undergo coronary angiography (79 vs. 70%) and percutaneous coronary intervention (PCI; 67 vs. 53%). When considering the subgroup of patients <80 years of age, the proportions of patients undergoing coronary angiography were much higher, and the difference between OHCA and AMI without CA was almost abolished (*Table 2*). In the subgroup of OHCA patients diagnosed with STEMI (*n* = 2156; *Table 2*), 1881 (87.2%) patients underwent coronary angiography, and 1733 (80.3%) were treated with PCI. Left ventricular ejection fraction (LVEF) was assessed by echocardiography before discharge in 77 846 (72%) patients. Acute myocardial infacrction patients with OHCA or IHCA were more likely to have LVEF below 40% compared with patients without CA (22, 35, and 10%, respectively).

**Table 2:** **Assessment and treatment of patients with myocardial infarction and no cardiac arrest, out-of-hospital cardiac arrest, and in-hospital cardiac arrest in Norway 2013–22^a^**I

	Myocardial infarction without cardiac arrest	Myocardial infarction with out-of-hospital cardiac arrest		Myocardial infarction with in-hospital cardiac arrest	
	*N* = 98 480	*N* = 3638	*P* *	*N* = 2559	*P* *
ST-elevation myocardial infarction (*n*)	25 505 (25.9%)	2156 (59.3%)	<0.001	1368 (53.5%)	<0.001
Pre-hospital fibrinolytic therapy	2089 (2.1%)	299 (8.2%)	<0.001	77 (3.0%)	0.002
Mean systolic blood pressure [mmHg (SD)] at hospital admission	142.2 ± 28.3	120.4 ± 32.7	<0.001	124.7 ± 31.7	<0.001
Mean diastolic blood pressure [mmHg (SD)] at hospital admission	80.8 ± 16.6	72.8 ± 21.0	<0.001	74.8 ± 20.0	<0.001
Coronary angiography (*n*)	68 735 (69.8%)	2874 (79.0%)	<0.001	1771 (69.2%)	0.52
Coronary angiography, age <80 years (*n*)	58 863/69 482 (84.7%)	2607/3018 (86.4%)	0.013	1454/1848 (78.7%)	<0.001
Coronary angiography, age ≥80 years (*n*)	9872/28 998 (34.0%)	267/620 (43.1%)	<0.001	317/711 (44.6%)	<0.001
PCI (*n*)	52 100 (52.9%)	2439 (67.0%)	<0.001	1518 (59.3%)	<0.001
Median delay from symptom onset to PCI (hours, IQR) in patients with ST-elevation myocardial infarction	3.9 (2.4, 8.8)	2.5 (1.8, 4.3)	<0.001	4.0 (2.3, 9.0)	0.66
LVEF ≤40% (*n*)	9833 (10.0%)	790 (21.7%)	<0.001	882 (34.5%)	<0.001
In-hospital cardiopulmonary resuscitation (*n*)	NA	907 (24.9%)	NA	NA	NA
Intra-aortic balloon pump (*n*)	554 (0.6%)	406 (11.2%)	<0.001	364 (14.2%)	<0.001
Respiratory support (*n*)	5135 (5.2%)	1632 (44.9%)	<0.001	924 (36.1.4%)	<0.001

Data are from the Norwegian Myocardial Infarction Registry.

SD, standard deviation; IQR, inter-quartile range; PCI, percutaneous coronary intervention; NA, not available.

^a^In patients with more than one myocardial infarction during the study period, only data from the first myocardial infarction are reported.

^*^Reference: patients with myocardial infarction without cardiac arrest.

### In-hospital complications and mortality

The rate of in-hospital complications was higher in AMI patients with OHCA/IHCA compared with AMI patients without CA (*Table 3*). Approximately one of three patients with IHCA developed cardiogenic shock, and 70% of these patients died.

**Table 3:** Complications during index hospitalization in patients with myocardial infarction and no cardiac arrest, out-of-hospital cardiac arrest, and in-hospital cardiac arrest in Norway 2013–22^a^

	Myocardial infarction without cardiac arrest	Myocardial infarction with out-of-hospital cardiac arrest		Myocardial infarction with in-hospital cardiac arrest	
	*N* = 98 480	*N* = 3638	*P* *	*N* = 2559	*P* *
Cardiogenic shock (*n*)	1844 (1.9%)	658 (18.1%)	<0.001	806 (31.5%)	<0.001
Mechanical complications (*n*)	371 (0.4%)	32 (0.9%)	<0.001	155 (6.1%)	<0.001
Stroke (*n*)	378 (0.4%)	35 (1.0%)	<0.001	12 (0.5%)	0.49
New myocardial infarction (*n*)	1289 (1.3%)	51 (1.4%)	0.63	140 (5.5%)	<0.001

Data are from the Norwegian Myocardial Infarction Registry.

^a^In patients with more than one myocardial infarction during the study period, only data from the first myocardial infarction are reported.

^*^Reference: patients with myocardial infarction without cardiac arrest.

A total of 4544 (4.6%) patients without CA, 1034 (28.4%) patients with OHCA, and 1260 (49.2%) patients with IHCA died during index hospitalization. As expected, the in-hospital mortality was significantly higher in patients with LVEF <40% compared with patients with LVEF ≥40%; 13.4 vs. 3.6% (*P* < 0.001) in patients without CA, 49.8 vs. 22.5% (*P* < 0.001) in patients with OHCA, and 60.7 vs. 43.2% (*P* < 0.001) in patients with IHCA.

### Long-term mortality

The median follow-up time was 3.3 (25th, 75th percentile: 1.1, 6.3) years. From admission to end of follow-up, a total of 32 505 (33.0%) patients without CA, 1578 (43.4%) patients with OHCA, and 1649 (64.4%) patients with IHCA died. The estimated cumulative 1-year mortality was 16% (95% CI: 15–16%) in AMI patients without CA, 36% (95% CI: 35–38%) in OHCA patients, and 57% (95% CI: 55–59%) in IHCA patients, and the estimated 5-year cumulative mortality was 35% (95% CI 34–35%), 48% (95% CI 46–50%), and 69% (95% CI 67–71%), respectively.

**Figure 3:**
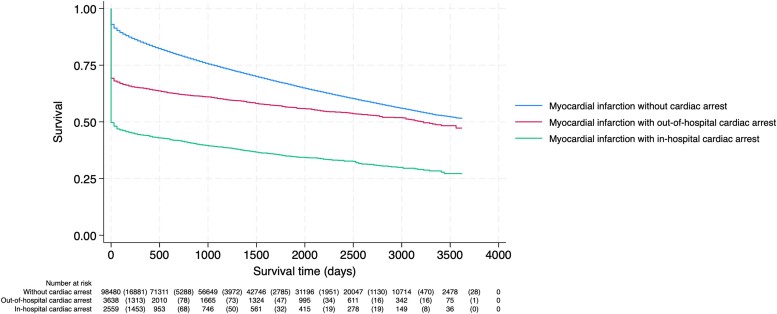
Kaplan–Meier curve of survival time in acute myocardial infarction patients without cardiac arrest, with out-of-hospital cardiac arrest, and with in-hospital cardiac arrest in Norway, 2013–22. Data are from the Norwegian Myocardial Infarction Registry. In patients with more than one acute myocardial infarction during the study period, only the first acute myocardial infarction is included.

*Figure 3* shows the Kaplan–Meier survival curve for the three groups of patients. The long-term risk of mortality from admission to end of follow-up in patients with OHCA and IHCA were significantly higher compared with AMI patients without CA [adjusted HR 2.35 (95% CI 2.24–2.48, *P* < 0.001) and 3.53 (95% CI 3.36–3.71, *P* < 0.001), respectively].

**Figure 4:**
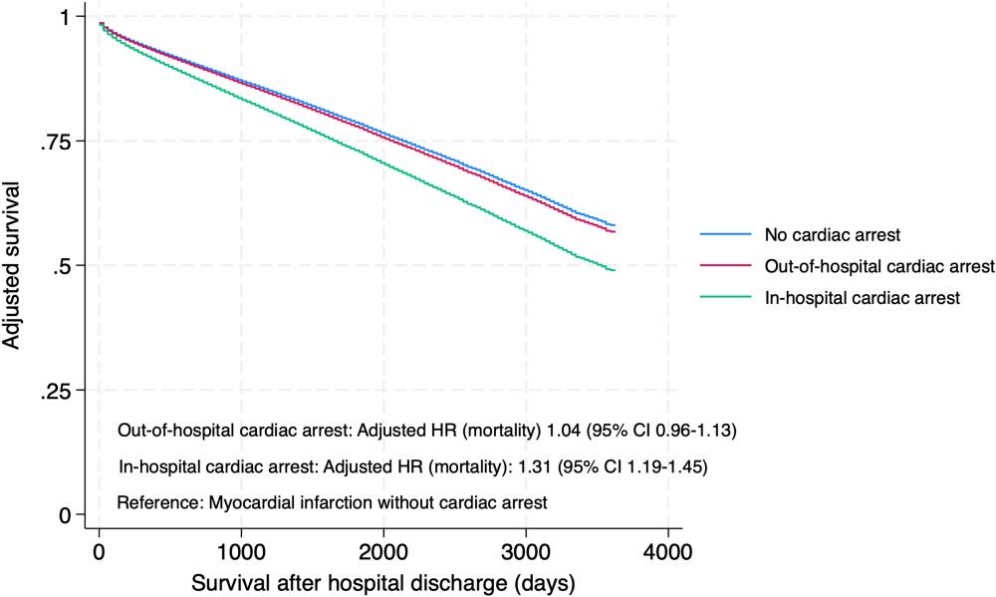
Adjusted* long-term survival of acute myocardial infarction patients surviving until hospital discharge (landmark analysis). Data are from the Norwegian Myocardial Infarction Registry, 2013–22. In patients with more than one acute myocardial infarction during the study period, only the first acute myocardial infarction is included. *Age, sex, smoking, previous acute myocardial infarction, previous stroke, previous heart failure, diabetes, antihypertensive treatment, and renal failure (eGFR < 60 mL/min/1.73 m^2^).

In AMI patients surviving to hospital discharge, the long-term risk of mortality did not differ between OHCA patients and patients without CA (adjusted HR 1.04, 95% CI 0.96–1.13, *P* = 0.341; *Figure 4*). However, patients with IHCA surviving to hospital discharge had higher long-term risk of mortality compared with AMI patients without CA (adjusted HR 1.31, 95% CI 1.19–1.45, *P* < 0.001).

In AMI patients who died after hospital discharge, ischaemic heart disease (International classification of diseases, tenth revision code I20-I25) was reported as the cause of death in 24, 32, and 34% of deaths in AMI without CA, AMI with OHCA, and AMI with IHCA, respectively.

#### Subgroup of patients <80 years and ≥80 years

**Figure 5:**
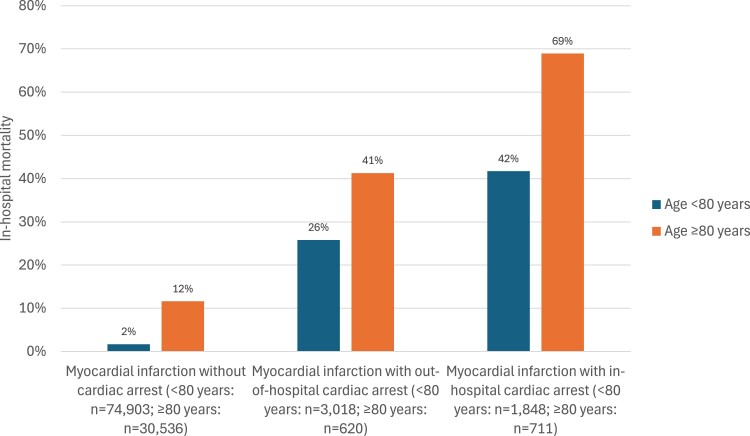
In-hospital mortality in patients with acute myocardial infarction who were <80 vs. ≥80 years, Norway 2013–22.

The in-hospital mortality rates in patients <80 years vs. ≥80 years are presented in *Figure 5*, and the estimated cumulative 30-day, 1-year, and 5-year mortality for patients <80 years and ≥80 years are given in *Table 4*. The adjusted HRs for long-term mortality in OHCA patients compared with AMIs without CA was similar across age groups (*Table 5*). For IHCA compared with AMIs without CA, a significant heterogeneity across age groups was shown (*Table 5*). However, due to the low number of IHCA patients ≥80 years, the findings must be interpreted with caution.

**Table 4:** Estimated cumulative mortality (life-table method) in patients <80 years and ≥80 years with myocardial infarction and no cardiac arrest, out-of-hospital cardiac arrest, and in-hospital cardiac arrest in Norway 2013–22^a^

	Myocardial infarction without cardiac arrest		Myocardial infarction with out-of-hospital cardiac arrest		Myocardial infarction with in-hospital cardiac arrest	
	<80 years	≥80 years	<80 years	≥80 years	<80 years	≥80 years
	*n* = 69 482	*n* = 29 998	*n* = 3018	*n* = 620	*n* = 1848	*n* = 711
30-day mortality (including in-hospital mortality) [% (CI)]	2.6 (2.5–2,8)	17.6 (17.2–18.0)	27.7 (26.2–29.4)	46.4 (42.5–50.4)	42.8 (40.6–45.1)	70.4 (67.0–73.7)
1-year post-discharge mortality (excluding in-hospital mortality) [% (CI)]	5.2 (5.1–5.4)	27.4 (26.9–28.0)	6.7 (5.7–7.9)	27.6 (23.3–32.6)	10.5 (8.8–12.6)	27.6 (22.1–34.2)
5-year post-discharge mortality (excluding in-hospital mortality) [% (CI)]	16.2 (15.9–16.5)	65.2 (64.6–65.8)	15.4 (13.8–17.2)	59.5 (54.2–65.0)	25.0 (22.3–28.0)	63.5 (56.7–70.3)

Data are from the Norwegian Myocardial Infarction Registry.

CI, confidence interval.

^a^In patients with more than one myocardial infarction during the study period, only data from the first myocardial infarction are reported.

**Table 5:** **Long-time mortality in patients <80 years and ≥80 years with myocardial infarction and no cardiac arrest, out-of-hospital cardiac arrest, and in-hospital cardiac arrest, surviving until hospital discharge, in Norway 2013–22** ^a^

	Myocardial infarction without cardiac arrest		Myocardial infarction with out-of-hospital cardiac arrest							Myocardial infarction with in-hospital cardiac arrest						
				Hazard ratio (95% CI)		Interaction *P*-value	Adjusted hazard ratio^b^ (95% CI)		Interaction *P*-value		Hazard ratio (95% CI)		Interaction *P-*value	Adjusted hazard ratio^b^ (95% CI)		Interaction *P*-value
	*n*		*n*							*n*						
<80 years	68 300	Reference	1078	0.90	(0.81–1.01)	0.671	0.98	(0.87–1.09)	0.550	1078	1.55	(1.37–1.75)	<0.001	1.37	(1.21–1.56)	<0.001
≥80 years	25 636	Reference	364	0.87	(0.76–0.99)		0.86	(0.75–0.98)		221	0.95	(0.81–1.13)		0.90	(0.76–1.07)	

Data are from the Norwegian Myocardial Infarction Registry.

CI, confidence interval.

^a^In patients with more than one myocardial infarction during the study period, only data from the first myocardial infarction are reported.

^b^Sex, smoking, previous myocardial infarction, previous stroke, previous heart failure, diabetes, antihypertensive treatment, and renal failure (eGFR < 60 mL/min/1.73 m^2^).

## Discussion

In this nationwide cohort of 105 439 patients with AMI admitted to hospitals in Norway from 2013 to 2022, 3.5% of the AMIs were complicated by OHCA and 2.4% by IHCA. Both OHCA and IHCA were associated with high in-hospital mortality; for IHCA, in-hospital mortality was almost 50%. However, among patients who survived to hospital discharge, long-term mortality did not differ between OHCA patients and AMI patients without CA (adjusted HR 1.04, 95% CI 0.96–1.13, *P* = 0.341). High age was associated with high in-hospital mortality in all groups as expected, but in patients who survived to hospital discharge, long-term survival after CA was comparable with AMI patients without CA across age groups.

The main strength of this study is the large and unselected population encompassing nearly all patients hospitalized with AMI in Norway from 2013 to 2022, along with nearly complete follow-up. However, given its observational nature, this study is subject to several limitations, and the results should be interpreted with caution. First, the NORMI did not have complete coverage for all variables. The registry does not contain information regarding initial heart rhythm, time intervals from CA to CPR or from CA to ROSC, or proportion of patients arriving hospital comatose or awake. Furthermore, we did not have any information about post-discharge treatment including implantable cardioverter defibrillators or the quality of life of survivors. Second, only AMI patients who were admitted to hospitals were registered in the NORMI. Third, diagnosing a preceding AMI in patients with OHCA may be challenging. Ischaemic changes in ECG and troponin rise and fall can also be observed in CA from other causes due to global ischaemia during the arrest. ST-elevation in post-ROSC ECGs has lower specificity in CA patients, and a diagnosis of STEMI may be difficult in these patients. The numbers of registered AMI patients with OHCA in the NORMI correspond to ∼10% of all EMS-treated OHCA and ∼35% of all hospitalized patients with OHCA in Norway in the study period.^12^ Consequently, the exact incidence of OHCA as a complication to AMI, or the proportion of OHCA with AMI aetiology, could not be estimated.

The annual proportions of OHCA among patients with AMI during the study period were low and consistent with previous reports.^13–15^ Despite a decrease in the overall number of AMIs registered in NORMI per year, there was no significant change in the proportion of patients with OHCA over the study period.^3,4^

In general, the risk of CA increases with age and cardiovascular morbidity.^16,17^ In this study, AMI patients with OHCA were younger and less likely to have prior cardiovascular disease compared with AMI patients without CA. Furthermore, they were more likely to be smokers than patients without CA and to have ST-segment elevation in their ECG.^18,19^ These findings are in accordance with previous findings that STEMI is more frequent in younger patients and is more frequently complicated by CA than nSTEMI.^20,21^ These findings may partially explain why long-term prognosis in patients surviving to hospital discharge was comparable in AMI patients with or without OHCA.

Early restoration of coronary blood flow is essential to optimize myocardial salvage and to reduce mortality in patients with AMI.^22^ The guidelines from the European Society of Cardiology recommended coronary angiography in most patients with AMI, but immediate routine coronary angiography is not recommended in all haemodynamically stable patients with CA.^23^ The proportion undergoing coronary angiography and PCI in this study was lower than might be expected. It should be noted however that very elderly AMI patients were included in our study (29% of the patients were ≥80 years of age). When considering only AMI patients <80 years of age, the percentages undergoing coronary angiography were much higher (86% for OHCA, 85% for AMI without CA). Increasing comorbidity with increasing age, and consequently a higher risk of complications, might have influenced the choice of treatment strategy in the elderly patient group. Considering all age groups together, patients with AMI and OHCA were more likely to undergo coronary angiography and PCI compared with patients without CA, probably reflecting that patients with AMI and OHCA were younger than patients without CA and more likely to have ST-elevation in the ECG. Furthermore, most AMI patients without ST-elevation on admission have no clear indication of immediate coronary angiography and may die before angiography is performed. We have not investigated geographical differences in treatment strategy, but long distances to hospitals offering coronary angiography in many areas could also influence the choice of treatment.

The majority of hospitalized AMI patients with and without OHCA or IHCA died during the index hospitalization. This is in accordance with previous findings.^13,14,24^ Cardiogenic shock occurred in 18% of AMI patients with OHCA and 32% of patients with IHCA and might partly explain the high mortality, although anoxic brain injury is the most frequent cause of in-hospital death in patients with OHCA arriving comatose to the hospital.^25^

Cardiogenic shock in the setting of myocardial infarction is still associated with >30% in-hospital mortality, with minimal improvement over time.^26^

The relatively high mortality of our AMI population reflects the high age of the population and are comparable with other European countries with ongoing national myocardial infarction registries.^27,28^ Most deaths occurred in patients >80 years or in patients with CA. Short-term outcome in patients <80 years without CA was excellent with 2.6% 30-day mortality.

Long-term survival rate in AMI complicated by OHCA in the study period was comparable with previous reports.^13,29,30^ A smaller study from Oslo University Hospital Ullevaal included 404 AMI patients with OHCA from 2005 to 2011 and reported a 30-day survival of 63.4%, slightly lower than in this nationwide study from 2013 to 2022 (69.1%).^13^ We found no significant difference in long-term mortality between AMI patients with and without OHCA who survived to hospital discharge. The good long-term outcome in OHCA patients surviving to hospital discharge is in line with previous results from Oslo, Norway, demonstrating that 84% of OHCA patients discharged alive from hospital were alive 4 years after with good neurological outcome.^31^

Although the in-hospital mortality in AMI patients ≥80 years was significantly higher in patients with OHCA compared with patients without CA, no difference in long-term survival was found in patients surviving to hospital discharge. This is reassuring and encourages EMS personnel to start CPR in patients ≥80 years.

In-hospital cardiac arrest in general has received less attention in clinical research compared with OHCA.^32,33^ The hospital incidence and case-fatality rates of ventricular tachycardia and/or ventricular fibrillation in patients with AMI have declined over the last decades due to an increased rate of early revascularization, less use of pro-arrhythmic drugs, and more extensive heart rhythm monitoring.^34^ In the present study, patients with IHCA represented ∼40% of the total number of patients with CA. The in-hospital mortality was as high as 49%, and the estimated cumulative 1-year mortality was 57%. A systematic review encompassing over 1 million IHCA cases from 1992 to 2016 revealed an overall 1-year survival rate of only 13%.^35^ The DANARREST registry from Denmark reported 20% 1-year survival rate in patients with IHCA.^36^ However, the DANARREST registry included all patients with IHCA, and less than half of the patients with IHCA had presumed cardiac aetiology.

## Conclusions

This large nationwide cohort study provides novel insights into clinical characteristics and outcomes of patients with AMI complicated by OHCA or IHCA. Despite advancements in treatment, the immediate mortality in AMI patients with OHCA or IHCA remains substantial. However, patients with OHCA, including elderly patients ≥80 years, surviving to hospital discharge exhibit a favourable long-term survival rate comparable with AMI patients without CA. Challenges still persist in mitigating short- and long-term mortality after IHCA. Further research is warranted to optimize treatment strategies for these high-risk populations.

## Data Availability

The data underlying this article were provided by the Norwegian Institute of Public Health under license/by permission. Data will be shared on request to the corresponding author with permission of the Norwegian Institute of Public Health.
